# HIF-1α antagonizes p53-mediated apoptosis by triggering HIPK2 degradation

**DOI:** 10.18632/aging.100254

**Published:** 2011-01-18

**Authors:** Lavinia Nardinocchi, Rosa Puca, Gabriella D'Orazi

**Affiliations:** ^1^Department of Experimental Oncology, Molecular Oncogenesis Laboratory, National Cancer Institute “Regina Elena”, Rome, Italy; ^2^Department of Oncology and Experimental Medicine, University “G. d'Annunzio”, Chieti, Italy

**Keywords:** HIF-1α, HIPK2, zinc, proteasomal degradation, p53 transcriptional activity, p53Ser46

## Abstract

Many human diseases are characterized by the development of tissue hypoxia. Hypoxia-inducible factor (HIF) is a transcription factor that regulates fundamental cellular processes in response to changes in oxygen concentration, such as angiogenesis, survival, and alterations in metabolism. The levels of HIF-1α subunit are increased in most solid tumors not only by low oxygen but also by growth factors and oncogenes and correlate with patient prognosis and treatment failure. The link between HIF-1α and apoptosis, a major determinant of cancer progression and treatment outcome, is poorly understood. Here we show that HIF-1α protects against drug-induced apoptosis by antagonizing the function of the tumor suppressor p53. HIF-1α upregulation induced proteasomal degradation of homeodomain-interacting protein kinase-2 (HIPK2), the p53 apoptotic activator. Inhibition of HIF-1α by siRNA, HIF-1α-dominant negative or by zinc re-established the HIPK2 levels and the p53-mediated chemosensitivity in tumor cells. Our findings identify a novel circuitry between HIF-1α and p53, and provide a paradigm for HIPK2 dictating cell response to antitumor therapies.

## INTRODUCTION

HIPK2 is a potential tumor suppressor gene; it is a nuclear serine/threonine kinase that acts as co-repressors for transcription factors [1] and is considered a central switch in the targeting of cells toward apoptosis upon genotoxic stress by phosphorylating tumor suppressor p53 at serine 46 (Ser46) [2,3]. The p53Ser46 modification triggers irreversible apoptosis by determining p53-dependent promoter selection [4]. HIPK2 contributes to p53 apoptotic activation by inducing Ser46 phosphorylation but also lysine 382 (Lys382) acetylation [2,3,5]. Thus, we found that HIPK2, through repression of Nox1 gene, strongly regulates p53 acetylation/deacetylation balance that along with (p)Ser46 is important for full p53 apoptotic activity [6]. A major determinant of tumor progression and cancer therapy is the ability of cancer cells to activate apoptotic cell death, mainly through intact p53 function. Understanding how aberrant signalling within tumors can interfere with p53 apoptotic activation is therefore of particular importance. Although mutations in the p53 gene are detected in ~50% of human cancers, indirect mechanism also leads to p53 inactivation [7]. Previous work has shown that knock-down of HIPK2, mainly by siRNA, leads to loss of p53 function, reduced apoptotic drug-response and increased tumor progression [8]. Therefore, an intact HIPK2 function is crucial for the apoptotic activation of wtp53 in tumors.

A physiological condition that inhibits HIPK2 functions in solid tumor is hypoxia which is a hallmark of tumor progression and failure of tumor therapies. Hypoxia-induced ubiquitin ligases such as Siah2 [9] or p53 targets MDM2 [10] and Siah1 [11] trigger HIPK2 degradation strongly affecting drug-induced p53 apoptotic activity. The key molecule expressed under hypoxia is hypoxia inducible factor-1 (HIF-1), a heterodimeric transcription factor that consists of the HIF-1β subunit, constitutively expressed in cells, and the oxygen-sensitive HIF-1α subunit. Under normal oxygen levels, HIF-1α is hydroxylated at key proline residues facilitating interaction to von Hippel-Lindau protein (pVHL) which allows HIF-1α proteasomal degradation. Under hypoxic conditions, prolyl hydroxylation is inhibited, HIF-1α accumulates, dimerizes with HIF-1β forming the active HIF-1 complex for regulation of transcription of several genes involved in many aspects of cancer progression, including angiogenesis, metabolic adaptation, apoptosis resistance, invasion and metastasis [12]. HIF-1α synthesis and transactivation can also be activated by non-hypoxia-mediated mechanisms such as genetic alterations in a variety of cancer types. In this regard, we have shown that HIPK2 represses the HIF-1α transcription, thus, HIPK2 knock-down leads to HIF-1α upregulation with induction of a “constitutive hypoxic” phenotype [13]. Increased HIF-1α levels have been shown to be associated with increased resistance to conventional chemo- and radiotherapy in many solid tumors and play a negative role in patient prognosis. Thus, the downregulation of the activity of HIF-1α could have an immediate effect on its target genes contributing to blocking tumor angiogenesis, glycolysis and tumor growth and also improve the efficacy of classical therapies [14].

HIF-1α interacts with p53 and stimulates p53 transcriptional activity [15] although it antagonizes p53-mediated apoptosis [16]. Here we report a previously unknown regulatory circuitry between HIF-1α, HIPK2 and p53 apoptotic activity as the molecular mechanisms underlying HIPK2 regulation by HIF-1α have never been addressed.

## RESULTS AND DISCUSSION

**Figure 1.**
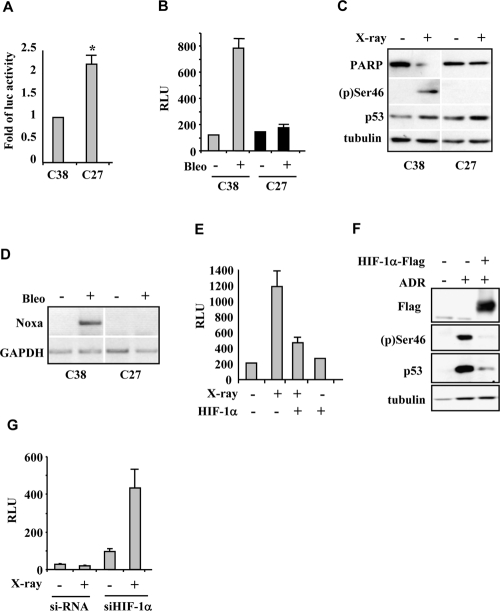
HIF-1α antagonizes p53 apoptotic activity. (**A**) Luciferase assay in C38 and C27 prostate cancer cells showed significantly higher HIF-1α-luc promoter activity in C27 cells. *P=0.0159. (**B**) Luciferase assay of p53AIP1-luc reporter in C38 and C27 prostate cancer cells after bleomycin (Bleo) treatment showing impairment of p53 transcriptional activity in C27 cells. (**C**) Immunoblot of C38 and C27 cells after 40 Gy X-irradiation revealed PARP cleavage and Ser46 phosphorylation only in C38 cells. Blot image was cut and pasted. (**D**) RT-PCR analysis of p53 apoptotic target genes in C38 and C27 prostate cancer cells after bleomycin (Bleo) treatment. Gel image was cut and pasted. (**E**) Luciferase assay of p53 target Noxa-luc reporter in C38 cells after X-irradiation revealed impairment of luciferase activity after HIF-1α overexpression. (**F**) Immunoblot of RKO cells after adriamycin (ADR) treatment showed abolishment of (p)Ser46 after HIF-1α-Flag overexpression. (**G**) Luciferase assay of p53AIP1-luc reporter in C27 cells showed induction of luciferase activity after HIF-1α silencing with siRNA.

**Figure 2.**
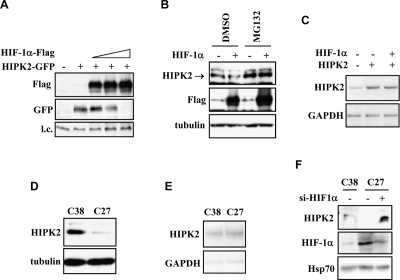
HIF-1α regulates HIPK2 protein degradation. (**A**) HIF-1α-Flag (4, 6, and 8 μg) and HIPK2-GFP (4 μg) expression vectors were co-transfected in 293 cells where immunoblot analyses showed that increased amounts of HIF-1α induced HIPK2 abolishment. (**B**) Immunoblot analysis of C38 cells transfected with HIF-1α expression and treated with proteasome inhibitor MG132 (10 μM for 4 h) or DMSO vehicle. The endogenous HIPK2 levels downregulated by HIF-1α were rescued by MG132. (**C**) RT-PCR analysis of HIPK2 in RKO colon cancer cells co-transfected with HIF-1α and HIPK2 expression vector. (**D**) Immunoblot of endogenous HIPK2 protein in C38 and C27 prostate cancer cells, showing lower HIPK2 levels in “constitutive hypoxic” C27 cells. (**E**) The HIPK2 mRNA levels were comparable between C38 and C27 cells. (**F**) Immunoblot in C27 cells showed rescue of endogenous HIPK2 protein levels after HIF-1α knock-down by siRNA.

We first analyzed the effect of HIF-1α on DNA-damage-induced p53 apoptotic activation by using an *ex vivo* experimental model consisting of cell populations derived from explants of prostate cancer patients characterized by stabilized HIF-1α protein in normoxia (“constitutively hypoxic” phenotype) and associated with bad prognosis (namely C27 cells), and cell populations with a phenotype negative for HIF-1α expression under aerobic condition associated with good prognosis (namely C38 cells) [17]. The presence of HIF-1α overexpression at mRNA (Figure 1A) and protein level (see Figure 2F) in C27 cells led to a marked inhibition of drug-induced luciferase activity of the p53AIP1 reporter gene (Figure 1B and Supplementary Figure 1a) which is a well established target of p53-Ser46 modification and of p53 apoptotic activity [4]. Thus, in response to X-ray or to the radiomimetic drug bleomycin, both Ser46 phosphorylation, the cleavage of the apoptotic marker PARP, and p53 apoptotic gene transcription were impaired in HIF-1α upregulated C27 cells, compared to C38 cells negative for HIF-1α expression under aerobic condition (Figure 1C, 1D). Two lines of evidence indicate that the p53 apoptotic defect in C27 cells is due to stabilization of HIF-1α rather than to alternative mechanism of drug resistance or impairment of p53 downstream signalling. First, increasing HIF-1α levels in C38 prostate and RKO colon cancer cells by protein overexpression also conferred resistance to X-ray- or to drug-induced p53 transcriptional activity (Figure 1E and Supplementary Figure S1b, S1c) and inhibited Ser46 phosphorylation (Figure 1F). Second, loss of HIF-1 function by HIF-1α knock-down, restored the sensitivity to X-ray-induced p53AIP1-luciferase activity in C27 cells (Figure 1G). These results show that HIF-1α levels are relevant to the p53-mediated cellular response because they antagonized drug-induced p53Ser46 apoptotic transcriptional activity.

P53Ser46 phosphorylation is triggered by several kinases including HIPK2 whose knock-down strongly inhibits p53 apoptotic activity [5,8]. Therefore, an intact HIPK2 function is crucial for the apoptotic activation of wtp53 in tumors. We first evaluated whether HIF-1α affected HIPK2 mRNA expression. RT-PCR analyses of ADR-treated RKO cells showed that endogenous HIPK2 messenger RNA levels were not altered by HIF-1α upregulation (Supplementary Figure S1c), although HIF-1α inhibited the drug-induced p53(p)Ser46 (Figure 1F), arguing for HIF-1α-mediated regulation of HIPK2 at the post-transcriptional level. We then performed experiments under conditions of HIF-1α and HIPK2 overexpression. Expression of increasing amounts of HIF-1α in 293 cells correlated with abolishment of HIPK2 protein levels (Figure 2A). A test for protein degradation showed that HIF-1α-induced HIPK2 downregulation in prostate C38 cells could be rescued by cell treatment with the proteasome inhibitor MG132 (Figure 2B), confirming a HIPK2 post-translational regulation. Thus, HIF-1α co-overexpression did not affect HIPK2 gene transcription in RKO colon cancer cells (Figure 2C). We next analysed these issues in C27 prostate cancer cells whereas HIF-1α upregulation antagonizes drug-induced p53Ser46 apoptotic transcriptional activity, suggesting that they should harbour reduced HIPK2 levels. Indeed, western blot analysis showed reduced HIPK2 protein levels in “constitutively hypoxic” C27 cells compared to the C38 cells with a phenotype negative for HIF-1α expression under aerobic condition (Figure 2D), while the HIPK2 mRNA levels were comparable expressed between the two cell lines (Figure 2E). Was the reduction of HIPK2 levels caused by HIF-1α upregulation? We addressed this issue by silencing of HIF-1α with siRNA that indeed rescued HIPK2 protein levels in C27 cells (Figure 2F). We thus conclude that HIF-1α regulates HIPK2 stability.

How could HIF-1α inhibit HIPK2? First, being a transcription factor, HIF-1 might promote the expression of target genes that induce HIPK2 degradation. Alternatively, HIF-1 might directly interact with and regulate HIPK2. To discriminate between these two scenarios, exogenous HIPK2 and HIF-1α proteins were co-expressed in 293 cells for co-immunoprecipitation analysis. We found absence of interaction between HIPK2 and HIF-1α (Supplementary Figure S2a), suggesting rather a transcription-dependent regulation. The latter hypothesis was evaluated by the use of a HIF-1α mutant encoding the dominant negative form of HIF-1α without DNA binding and trans-activation domains (HIF-1αDN) [18]. The results unequivocally showed that the HIF-1αDN mutant could not inhibit HIPK2 stability (Supplementary Figure S2b).

Previous studies showed that HIF-1 may induce p53 transcriptional activity [15], although not the apoptotic one [16], and that p53 target genes such as MDM2 [10] or Siah1 [11] may trigger HIPK2 degradation under hypoxia. Moreover, the putative HIF-1 target WD40-repeat/SOCS box protein WSB-1 [19] has been shown to trigger HIPK2 degradation [20]. To evaluate whether p53 transcriptional activity plays a role in HIF-1α-induced HIPK2 degradation, HIF-1α and HIPK2 proteins were co-expressed in H1299 lung cancer (p53 null) cells and assayed for western immunoblotting. In the absence of p53, HIF-1α was still able to reduce HIPK2 protein levels (Supplementary Figure S2c). We conclude that HIF-1α inhibits HIPK2 *via* transcriptional upregulation of one or more target genes.

**Figure 3.**
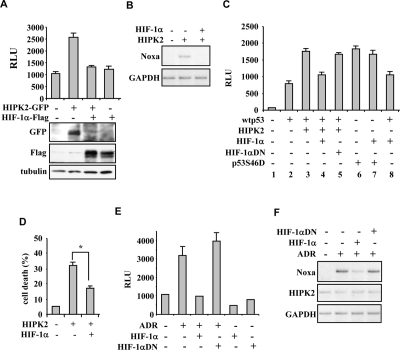
HIPK2-induced p53 apoptotic activity is impaired by HIF-1α. (**A**) Luciferase assay in 293 cells co-transfected with Noxa-luc reporter and HIPK2-GFP (4 μg) expression vector alone or in combination with HIF-1α-Flag (8 μg). Results represent mean ± s.d. from three experiments. The expression of the ectopic proteins was assayed by immunoblot. (**B**) Lysates from RKO cells co-transfected with HIF-1α and HIPK2 expression vectors were assayed for RT-PCR of p53 target gene Noxa. GAPDH is a loading control (**C**) Luciferase assay in H1299 cells co-transfected with Noxa-luc reporter and low amount of wtp53 expression vector or of p53S46D mutant, in combination with HIPK2, HIF-1α or HIF-1αDN mutant. Results represent mean ± s.d. from three experiments. (**D**) Tunel assay of RKO cells where HIF-1α overexpression significantly reduced the HIPK2-induced apoptotic cell death. *P=0.001. (**E**) Luciferase assay in RKO cells stable transfected with p53AIP1-luc reporter where the adryamicin (ADR)-induced luciferase activity was inhibited by HIF-1α overexpression but not by HIF-1α dominat negative (DN) mutant. Results represent mean ± s.d. from three experiments. (**F**) Lysates from RKO cells treated as indicated were assayed for RT-PCR analyses of p53 apoptotic target Noxa and for HIPK2 expression. GAPDH is a loading control.

The above results demonstrate that HIF-1α upregulation reduces both HIPK2 protein levels and p53 apoptotic activation. The direct effect of HIF-1α on HIPK2-induced p53 activation was then analysed under conditions of proteins overexpression. The luciferase assay performed in 293 cells clearly showed that the positive effect of HIPK2 on endogenous p53 apoptotic transcriptional activity was eliminated by HIF-1α co-expression that indeed abolished HIPK2 levels (Figure 3A). The abolishment of p53 transcriptional activity was confirmed *in vivo* in RKO cells by RT-PCR analysis (Figure 3B). We then performed experiments with p53 overexpression in H1299 cells. HIF-1α abolished the HIPK2 additive effect on p53-induced Noxa-luc activity (Figure 3C, compare lane 3 with lane 2 and lane 4 with lane 3), while HIF-1αDN mutant failed to do so (Figure 3C, compare lane 5 with lane 4), suggesting that HIF-1α is acting on HIPK2 function. To finally demonstrate this issue, the transcriptional activity of a p53S46D mutant, which expresses constitutive Ser46 phosphorylation, was not inhibited by HIF-1α (Figure 3C, compare lane 7 with lane 6), confirming that HIPK2 is indeed the target of HIF-1α-induced p53 regulation. Next, we investigated the effect of HIPK2 inhibition on apoptosis. The results clearly showed that HIF-1α significantly counteracted HIPK2-induced cell death (Figure 3D). Finally, HIF-1α overexpression led to marked reduction of ADR-induced p53AIP1-luc activity in RKO cells, while the HIF-1α-DN mutant did not show such effect (Figure 3E), as also tested by *in vivo* by RT-PCR analyses (Figure 3F). These results recapitulate the negative effect of constitutive hypoxic phenotype on HIPK2-induced p53 apoptotic transcriptional activity and cell response to drug.

**Figure 4.**
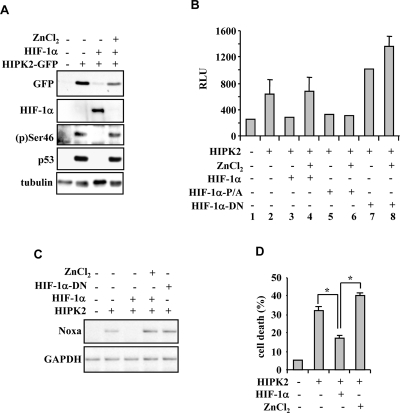
Zinc reactivates the HIF-1α-inhibited HIPK2/p53 signalling. (**A**) Immunoblot of RKO cells in which the effect of HIPK2 overexpression on p53Ser46 phosphorylation was abolished by HIF-1α co-expression and restored by concomitant zinc (100 μm for 24 h) treatment. (**B**) Luciferase assay in RKO cells stable transfected with p53AIP1-luc reporter showing that the HIPK2-induced luciferase activity was inhibited by HIF-1α and rescued by zinc treatment (100 μm for 24 h); zinc did not rescued the HIPK2 inhibition triggered by the HIF-1αP/A mutant and the HIF-1αDNA mutant did not inhibit HIPK2-induced transcriptional activity. Results represent mean ± s.d. from three experiments. (**C**) Lysates from RKO cells treated as indicated were assayed for RT-PCR analyses of p53 apoptotic target Noxa. GAPDH is a loading control. (**D**) Tunel assay of RKO cells showing that the HIPK2-induced apoptotic cell death was significantly inhibited by HIF-1α and strongly rescued by zinc treatment. *P=0.001.

We recently reported that zinc inhibits HIF-1α stability *in vitro* and *in vivo* by acting on prolyl hydroxylation and VHL interaction [21]. We also showed that zinc inhibits MDM2 ubiquitin ligase activity, counteracting MDM2-induced p53 degradation and re-establishing HIPK2 stability [22]. Restoration of wtp53 function is decisive for the success of anti-tumor therapies and for tumor regression *in vivo* [23,24]. Therefore, zinc treatment of tumor cells with HIF-1α upregulation and that retain wild-type p53 should result in (*i*) reconstitution of HIPK2-induced p53Se46 phosphorylation, (*ii*) activation of p53-apoptotic genes and (*iii*) restoration of drug-induced apoptosis. The HIPK2-induced p53(p)Ser46 in RKO cells, abolished by HIF-1α co-expression, was completely re-established by zinc treatment that, as expected, concomitantly rescued the HIF-1α-induced HIPK2 downregulation (Figure 4A). The HIPK2-induced p53 transcription of p53AIP1-luc reporter, inhibited by HIF-1α, was strongly recovered by zinc treatment (Figure 4B, compare lane 4 with lane 3), while the inhibition triggered by a HIF-1α expression vector with prolyl mutations P402A and P564A (HIF-1αP/A) was not rescued by zinc (Figure 4B, compare lane 6 with lane 5), in agreement with our findings that the HIF-1αP/A mutant is not downregulated by zinc [21]; finally, the HIF-1αDN mutant did not inhibit the HIPK2-induced p53AIP1-luc activity (Figure 4B, compare lane 7 with lanes 5 or 3) that was rather superinduced by zinc (Figure 4B, compare lane 8 with lane 7), suggesting inhibition of endogenous HIF-1α by the HIF-1αDN mutant. The positive effect of zinc on rescue of HIPK2/p53 activity in the presence of HIF-1α upregulation was confirmed by *in vivo* RT-PCR analysis of p53 apoptotic target genes (Figure 4C) and by cell death analysis (Figure 4D).

The above results demonstrate that zinc treatment counteracts the HIF-1α-mediated inhibition of HIPK2, allowing restoration of p53 response to antitumor therapies. Thus, this issue was addressed in C27 prostate cancer cells in which both HIPK2 and p53 are disabled by constitutive HIF-1α upregulation. The results clearly showed that zinc restored (*i*) the endogenous p53 transcriptional activity in response to X-irradiation, (*ii*) the *in vivo* transcription of p53 target genes in response to drug and (*iii*) the cell death to genotoxicity (Supplementary Figure S3a-c), again implicating the HIF-1α/HIPK2 circuitry as shown by rescue of endogenous HIPK2 stability by zinc (Supplementary Figure S3d). These results are consistent with several observations by us showing that zinc supplementation can increase tumor response to drugs and counteract the negative effect of hypoxia, by acting on several interconnected signalling molecules such as HIPK2, HIF-1 and p53 [25].

Restoration of HIPK2 activity was finally monitored by chromatin immunoprecipitation assay as HIPK2 is able to modulate the transcription of several factors involved in cell survival and apoptosis [1]. The results showed that HIPK2 recruitment onto target genes such as *Bcl-2* [26] or *CYP1B1* [9] was present in basal condition only in C38 cells compared to the C27 cells where it was instead rescued by zinc treatment (Supplementary Figure S4). HIPK2 recruitment onto *Bcl-2* promoter is again indicative of functioning p53 as HIPK2 has been shown to be involved in the transcriptional repression of *Bcl-2* promoter exerted by p53 [26].

HIF-1α stabilization due to low oxygen or because genetic alterations is responsible of increased chemo-resistance and tumor cell viability in part due to inhibition of p53 apoptotic gene transcription [10]. The discovery here of the HIF-1α/HIPK2 circuitry gives a mechanistic explanation of the p53 apoptotic inhibition in response to drug under hypoxia in those tumors that retain a not functional wild-type p53. Thus, for the first time, it is shown a direct effect of HIF-1α on HIPK2 protein stability. Moreover, our findings also open novel and unexpected scenarios in tumor development and ask to whether HIPK2 might participate to the regulation of cancer stem cells (SCs). Hypoxia and HIFs-α subunits are considered a critical component of a cancer stem cell niche in different tumors including glioblastoma [27] and stem-like cells may be integral to the development and maintenance of human cancers [28]. Our data raise the possibility that HIPK2, because of the HIF-1α relationship is involved in the homeostasis of cancer SCs and in its subversion in tumors. Recent findings showed that loss of p53 favours symmetric divisions of cancer SCs, contributing to tumor growth [29]. As HIPK2 inhibition negatively affects p53 activity [8,30] it would be interesting to evaluate whether loss of p53 function in cancer SCs depends on HIF-1α-induced HIPK2 inhibition and propose an additional mechanism of tumorigenesis. Identification of the cell types capable of initiating and sustaining growth of the neoplastic clone *in vivo* is a fundamental problem in cancer research. Understanding the nature of the more quiescent cancer stem-like cells and their niches has the potential to development of novel cancer therapeutic protocols including pharmacological targeting of self-renewal pathways. Therefore, our data strengthen the notion that unleashing the growth suppressor activity of both HIPK2 and p53 by targeting HIF-1α with zinc is a potentially valuable adjuvant strategy for cancer treatment.

## METHODS

### Cell culture and treatments

Human patients-derived prostate cancer cell lines C38 and C27, [17,31] (kindly provided by A. Farsetti, National Research Council, Rome, Italy), human embryo kidney 293, were maintained in DMEM (Life Technology-Invitrogen), while human lung cancer H1299 (p53 null) and colon cancer RKO were maintained in RPMI-1640 (Life Technology-Invitrogen), all supplemented with 10% heat-inactivated fetal bovine serum plus glutamine and antibiotics.

Subconfluent cells were treated with adriamycin (ADR) diluted into the medium to a final concentration of 1.5 μg/ml, bleomycin (Bleo) diluted into the medium to a final concentration of 120 μM, zinc chloride (ZnCl_2_) diluted into the medium to a final concentration of 100 μM, or X-ray irradiated with 40 Gy, for the indicated period of time. Proteasome inhibitor MG132 at final concentration of 10 μM was added for 4 h.

### Western blotting and co-immunoprecipitation

Total cell extracts and nuclear extracts were prepared essentially as described [13]. Proteins were transferred to a polyvinylidene difluoride (PVDF) (Millipore) or nitrocellulose (Biorad) membranes. Immunoblottings were performed with the following antibodies: mouse monoclonal anti-HIF-1α, (Novus Biologicals), mouse monoclonal anti-p53 (DO1) (Santa Cruz Biotechnology), rabbit polyclonal anti-phospho-Ser46, (Cell Signaling Technology), rabbit polyclonal anti-HIPK2 (kindly provided by M.L. Schmitz, Justus-Liebig-University, Giessen, Germany), mouse monoclonal anti-poly(ADP-ribose) polymerase (PARP, BD Pharmingen), monoclonal anti-GFP (Roche Diagnostic), mouse monoclonal anti-Flag (Sigma), mouse monoclonal anti-tubulin (Immunological Sciences), and mouse monoclonal anti-Hsp70 (Stressgene). Immunoreactivity was detected by enhanced chemiluminescence kit (ECL; Amersham). For HIF-1α/HIPK2 co-immunoprecipitation 293 cells were co-transfected with 4 μg HIPK2-GFP and 8 μg HIF-1α-Flag expression vector for 24 h. Total cell extracts were prepared by incubating at 4^°^ C for 30 min in lysis buffer (20 mmol/L Hepes, 100 mmol/L NaCl, 5 mM EDTA (pH 8.0), 10% glicerol). Following preclearing for 1 h at 4^°^ C, immunoprecipitation was performed by incubating total cell extracts with anti-Flag antibody pre-adsorbed to protein G-Agarose (Pierce), rocking for 2 h at 4^°^ C. The beads were then resuspended in 5x Laemmli buffer and subjected to Western blot with the indicated primary antibodies.

### RNA extraction and reverse transcription (RT)-PCR analysis

Cells were harvested in TRIzol Reagent (Invitrogen) and total RNA was isolated following the manufacturer's instructions essentially as described [30]. PCR was performed by using gene specific oligonucleotides under conditions of linear amplification. PCR products were run on a 2% agarose gel and visualized by ethidium bromide staining using UV light. The housekeeping GAPDH mRNA was used as internal control.

### Transfection, plasmids and transactivation assay

Cells (RKO, C27 C38) were transfected with the cationic polymer LipofectaminePlus method (Invitrogen) according to manufacturers' instructions or (293 and H1299) with the N,N-bis-(2- hydroxyethyl)-2-amino-ethanesulphonic acid-buffered saline (BBS) version of the calcium phosphate procedure [32].

For luciferase activity the plasmid reporter used were: the HIF-1α-pH800-luc promoter (kindly provided by C. Michiels, FUNDP-University of Namur, Belgium), the p53-dependent promoters Noxa-luc (kindly provided by T. Taniguchi, University of Tokyo, Japan) and p53AIP1-luc (kindly provided by H. Arakawa, National Cancer Center, Tokyo, Japan). The amount of plasmid DNA in each sample was equalized by supplementing with empty vector. Transfection efficiency was normalized with the use of a co-transfected β-galactosidase (β-gal) plasmid. Luciferase activity was assayed on whole cell extract and the luciferase values were normalized to β-galactosidase activity and protein content and expressed as relative luciferase unit (RLU).

The expression vectors used were: the Flag-tagged HIF-1αand the Flag-tagged HIF-1α with prolyl mutations P402A and P564A [33] (kindly provided by G.L. Semenza, The Johns Hopkins University School of Medicine, Baltimore, MD, USA), the dominant negative form of HIF-1α without DNA binding domain and transactivation domain (pCEP4-HIF-1αDN) [18] (kindly provided by B.H. Jiang, Nanjing Medical University, China), HIPK2-GFP [2], pCMV-wtp53, and the p53Ser46D (constitutively phosphorylated) (kindly provided by Dr. L Mayo, Case Western Reserve University, Cleveland, Ohio, USA) mutant.

### siRNA interference

Cells were plated at semiconfluence in 35 mm dishes the day before transfection. Control-siRNA and siHIF-1α (Dharmacon) were transfected overnight using LipofectaminePlus reagent (Invitrogen) and 24 h later cells were trypsinized and replated and transfected for the indicated period before harvesting for luciferase activity.

### Chromatin Immunoprecipitation (ChIP) assay

Chromatin Immunoprecipitation (ChIP) analysis was carried out essentially as described [30]. Protein complexes were cross-linked to DNA in living cells by adding formaldehyde directly to the cell vulture medium at 1% final concentration. Chromatin extracts containing DNA fragments with an average size of 500 bp were incubated overnight at 4^°^ C with milk shaking using rabbit polyclonal anti-HIPK2 (Santa Cruz Biotechnology) antibody. Before use, protein G (Pierce) was blocked with 1 μg/μL sheared herring sperm DNA and 1 μg/μL BSA for 3 h at 4^°^ C and then incubated with chromatin and antibodies for 2 h at 4^°^ C. PCR was performed with HOT-MASTER Taq (Eppendorf) using 2 μL of immuniprecipitated DNA and promoter-specific primers for human *Bcl-2*[26], and *CYP1B1*[9] promoters. Immunoprecipitation with non-specific immunoglobulins (IgG; Santa Cruz Biotechnology) was performed as negative controls. The amount of precipitated chromatin measured in each PCR was normalized with the amount of chromatin present in the input of each immunoprecipitation. PCR products were run on a 2% agarose gel and visualized by ethidium bromide staining using UV light.

### TUNEL assay

For TUNEL assay, 4×10^4^ cells were spun on a slide by cytocentrifugation and subsequently fixed in 4 % paraformaldehyde for 30 min at room temperature. After rinsing with PBS the samples were permeabilized in a solution of 0.1 % Triton X-100 in sodium citrate for 2 min. Samples, washed with PBS, were then incubated in the TUNEL reaction mix for 1 h at 37^°^C, according to the manufacturer's instructions (Roche, Germany). Cells were counter-stained with Hoechst 33342 before analysis with a fluorescent microscope (Zeiss). Standard deviations of three independent experiments were indicated.

### Statistics

All experiment unless indicated were performed at least three times. All experimental results were expressed as the arithmetic mean and standard deviation (s.d.) of measurements was shown. Student's *t*-test was used for statistical significance of the differences between treatment groups. Statistical analysis was performed using analysis of variance at 5% (p<0.05) or 1% (p<0.01).

## SUPPLEMENTARY FIGURES

Figure S1. HIF-α confers resistance to drug-induced p53 apoptotic activation. (**a**) Luciferase assay showed impaired p53AIP1-luc activity in C27 cells in response to X-ray irradiation, compared to the C38 cells. Results represent mean ± s.d. from three experiments. (**b**) Luciferase assay of RKO cells stable transfected with p53AIP1-luc reporter where HIF-1α overexpression inhibited the adryamicin (ADR)-induced p53 transcriptional activity. Results represent mean ± s.d. from three experiments. (**c**) RT-PCR analysis of p53 apoptotic target genes in RKO colon cancer cells where HIF-1α overexpression inhibited the adryamicin (ADR)-induced p53 target gene transcription. GAPDH was a loading control.

Figure S2. HIF-1α inhibits HIPK2 through its transcriptional activity. (**a**) 293 cells were co-transfected with 4 μg HIPK2-GFP and 8 μg HIF-1α-Flag and 24 h after later equal amount of total cell extracts were immune-precipitated with anti-Flag antibody and immunoblotted with anti-GFP antibody to detect protein/protein interaction. Input is 1/10 of the total cell extracts used for immune-precipitation. (**b**) Immunoblot of 293 cells co-transfected with HIPK2-GFP (4 μg) alone or in combination with the HIF-1αDN (8 μg) expression vectors. The HIPK2 protein levels were not abolished by HIF-1αDN. Anti-tubulin was used as protein loading control. (**c**) Immunoblot in H1299 cells (p53 null) co-transfected as in (**b**). The HIPK2 protein levels were strongly abolished by HIF-1α. Anti-tubulin was used as protein loading control.

Figure S3. Zinc restores p53 activity in HIF-1α-upregulated cells. (**a**) Luciferase assay showed that the impaired Noxa-luc activity in C27 cells in response to X-ray irradiation was counteracted by zinc treatment. Results represent mean ± s.d. from three experiments. (**b**) Similar result was obtained in C27 cells by RT-PCR analysis where zinc restored the p53 apoptotic gene transcription in response to bleomycin (Bleo). GAPDH was used as internal control. (**c**) Tunel assay of C27 cells showing increased apoptotic cell death only after zinc supplementation to Bleo treatment. (**d**) Immunoblot showing increased endogenous HIPK2 levels in C27 after zinc treatment. Anti-tubulin was used as protein loading control.

Figure S4. Zinc restores HIPK2 recruitment onto target promoter in HIF-1α-upregulated cells. Chromatin immunoprecipitation (ChIP) analysis performed with anti-HIPK2 antibody on C38 cells and C27 cells untreated or treated with zinc (100 μM for 24 h). PCR analyses were performed on the immunoprecipitated DNA samples using specific primers for the human Bcl-2 and CYP1B1 gene promoters. A sample representing linear amplification of the total input chromatin (Input) was included as control. Additional controls included immunoprecipitation performed with non-specific immunoglobulins (No Ab).
