# Supplementary figures and images for: HIF-1α antagonizes p53-mediated apoptosis by triggering HIPK2 degradation
Source: Aging (Albany NY). 2011 Jan 18;3(1):33–43. doi: 10.18632/aging.100254 (PMC3047137; doi:10.18632/aging.100254)

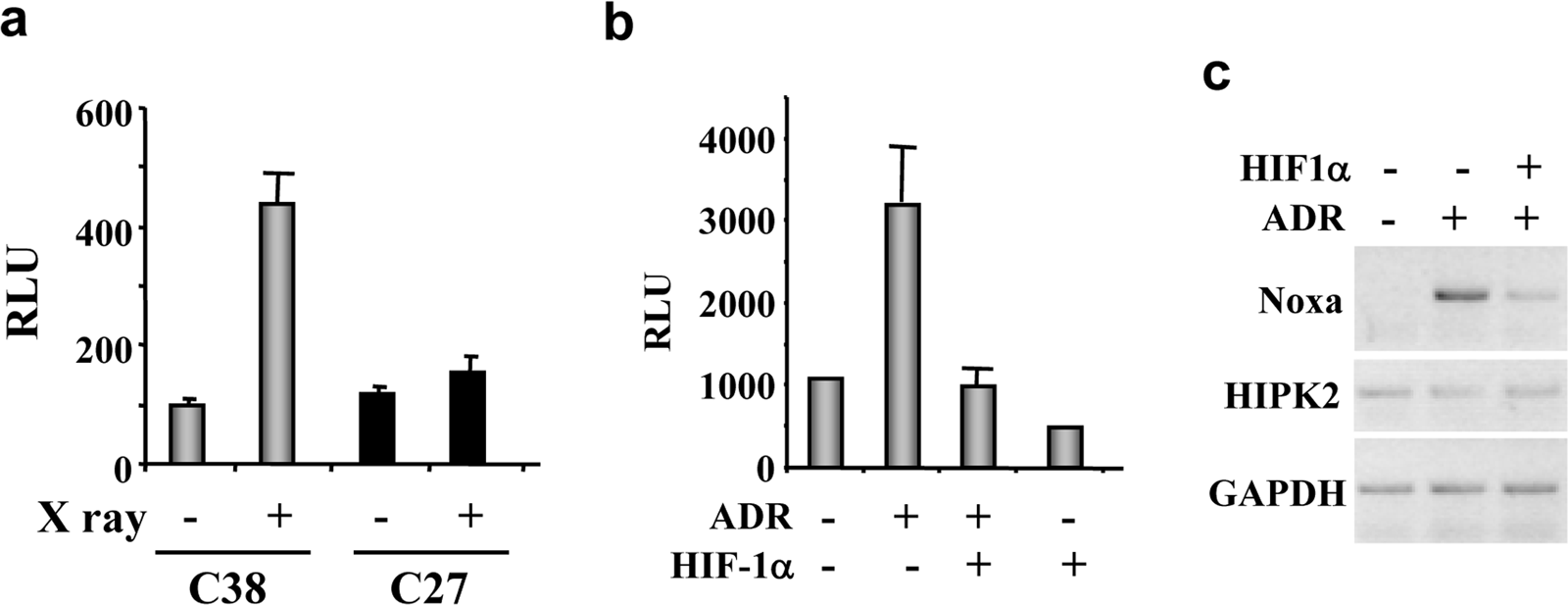

Supplement: Figure S1. — (a) Luciferase assay showed impaired p53AIP1-luc activity in C27 cells in response to X-ray irradiation, compared to the C38 cells. Results represent mean ± s.d. from three experiments. (b) Luciferase assay of RKO cells stable transfected with p53AIP1-luc reporter where HIF-1α overexpression inhibited the adryamicin (ADR)-induced p53 transcriptional activity. Results represent mean ± s.d. from three experiments. (c) RT-PCR analysis of p53 apoptotic target genes in RKO colon cancer cells where HIF-1α overexpression inhibited the adryamicin (ADR)-induced p53 target gene transcription. GAPDH was a loading control. [file aging-03-33-s001.tif]

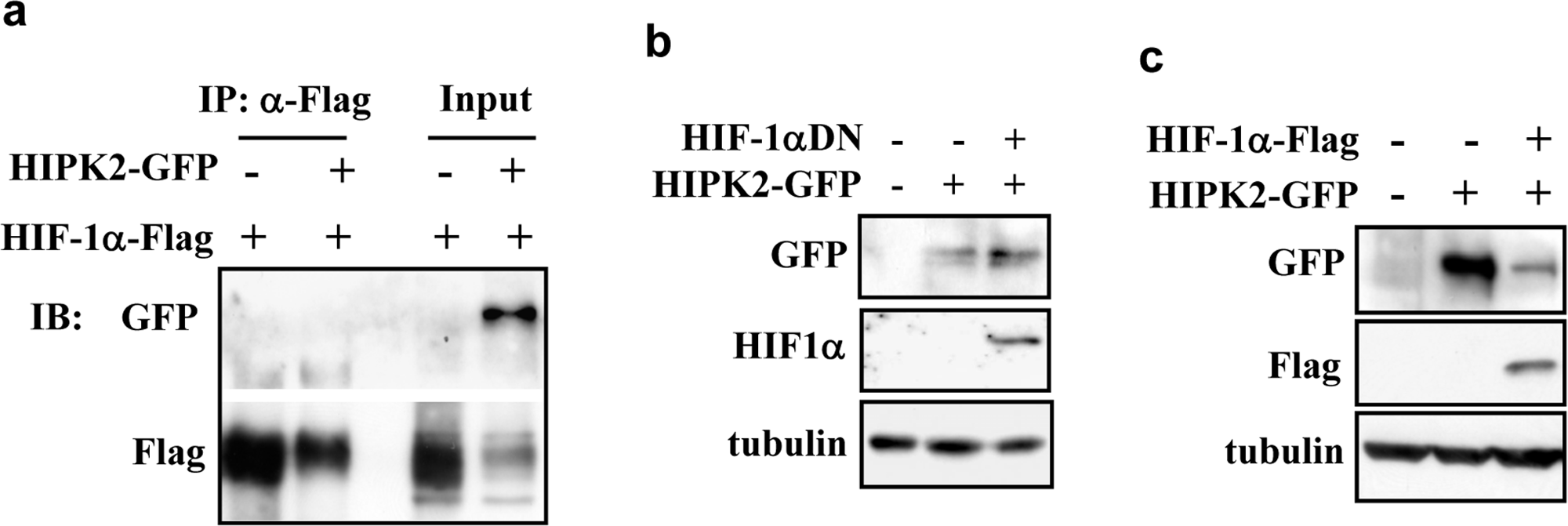

Supplement: Figure S2. — (a) 293 cells were co-transfected with 4 μg HIPK2-GFP and 8 μg HIF-1α-Flag and 24 h after later equal amount of total cell extracts were immune-precipitated with anti-Flag antibody and immunoblotted with anti-GFP antibody to detect protein/protein interaction. Input is 1/10 of the total cell extracts used for immune-precipitation. (b) Immunoblot of 293 cells co-transfected with HIPK2-GFP (4 μg) alone or in combination with the HIF-1αDN (8 μg) expression vectors. The HIPK2 protein levels were not abolished by HIF-1αDN. Anti-tubulin was used as protein loading control. (c) Immunoblot in H1299 cells (p53 null) co-transfected as in (b). The HIPK2 protein levels were strongly abolished by HIF-1α. Anti-tubulin was used as protein loading control. [file aging-03-33-s002.tif]

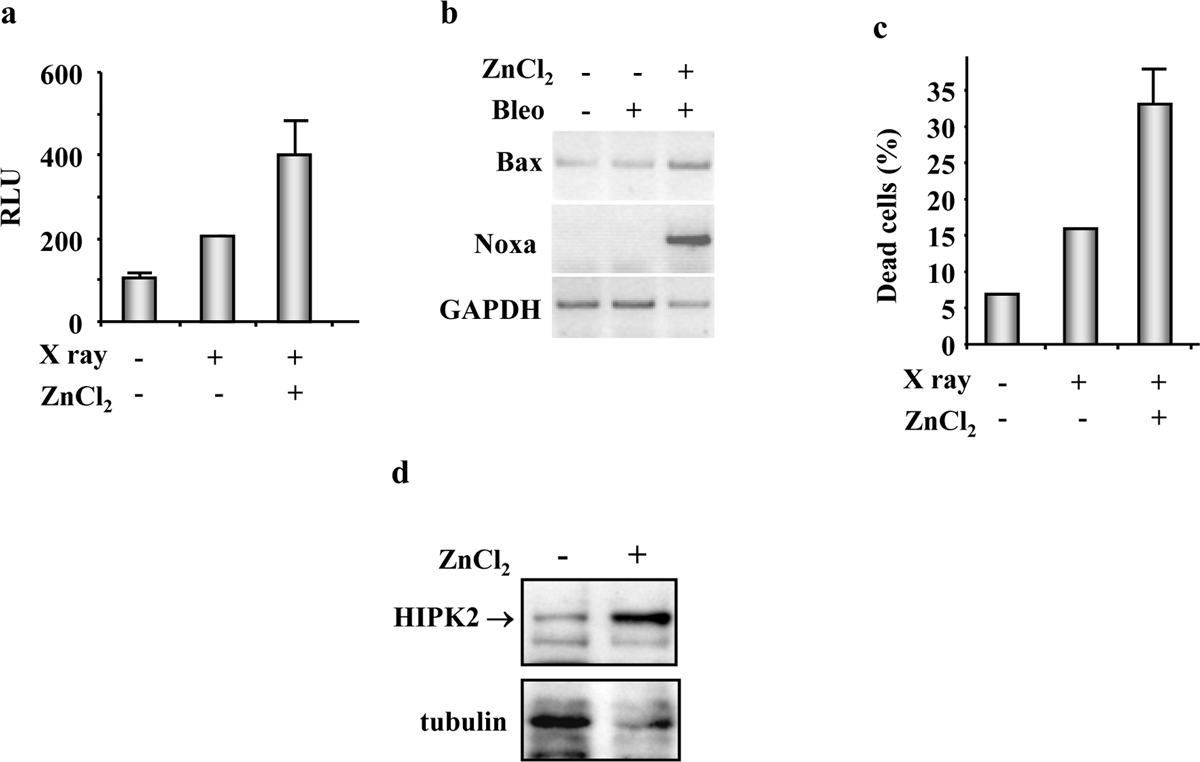

Supplement: Figure S3. — (a) Luciferase assay showed that the impaired Noxa-luc activity in C27 cells in response to X-ray irradiation was counteracted by zinc treatment. Results represent mean ± s.d. from three experiments. (b) Similar result was obtained in C27 cells by RT-PCR analysis where zinc restored the p53 apoptotic gene transcription in response to bleomycin (Bleo). GAPDH was used as internal control. (c) Tunel assay of C27 cells showing increased apoptotic cell death only after zinc supplementation to Bleo treatment. (d) Immunoblot showing increased endogenous HIPK2 levels in C27 after zinc treatment. Anti-tubulin was used as protein loading control. [file aging-03-33-s003.tif]

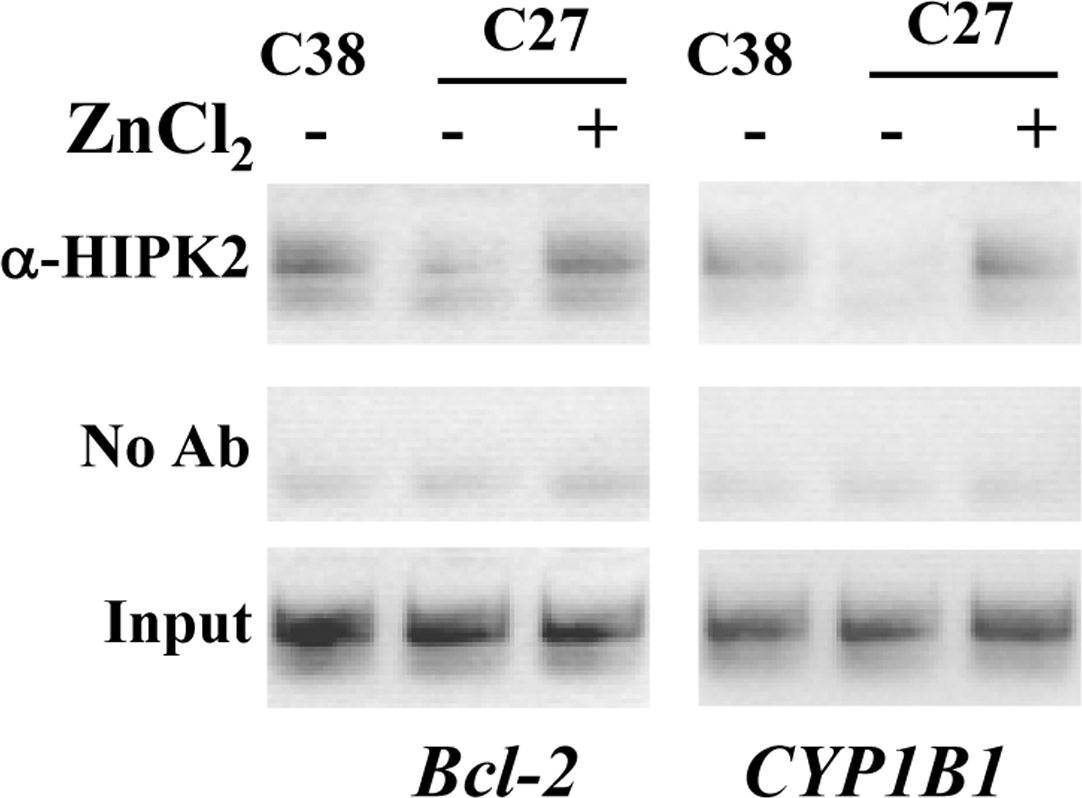

Supplement: Figure S4. — Chromatin immunoprecipitation (ChIP) analysis performed with anti-HIPK2 antibody on C38 cells and C27 cells untreated or treated with zinc (100 μM for 24 h). PCR analyses were performed on the immunoprecipitated DNA samples using specific primers for the human Bcl-2 and CYP1B1 gene promoters. A sample representing linear amplification of the total input chromatin (Input) was included as control. Additional controls included immunoprecipitation performed with non-specific immunoglobulins (No Ab). [file aging-03-33-s004.tif]
